# Evolution of HER2-positive mammary carcinoma: HER2 loss reveals claudin-low traits in cancer progression

**DOI:** 10.1038/s41389-021-00360-9

**Published:** 2021-11-13

**Authors:** Veronica Giusti, Francesca Ruzzi, Lorena Landuzzi, Marianna L. Ianzano, Roberta Laranga, Elena Nironi, Laura Scalambra, Giordano Nicoletti, Carla De Giovanni, Martina Olivero, Maddalena Arigoni, Raffaele Calogero, Patrizia Nanni, Arianna Palladini, Pier-Luigi Lollini

**Affiliations:** 1 Laboratory of Experimental Oncology, IRCCS Istituto Ortopedico Rizzoli, Bologna, Italy; 2 Laboratory of Immunology and Biology of Metastasis, Department of Experimental, Diagnostic and Specialty Medicine (DIMES), University of Bologna, Bologna, Italy; 3 Candiolo Cancer Institute-FPO, IRCCS, Candiolo, Torino, Italy; 4 Department of Molecular Biotechnology and Health Science, University of Torino, Torino, Italy; 5 Department of Pharmacy and Biotechnology, University of Bologna, Bologna, Italy

**Keywords:** Breast cancer, Tumour heterogeneity

## Abstract

HER2-positive breast cancers may lose HER2 expression in recurrences and metastases. In this work, we studied cell lines derived from two transgenic mammary tumors driven by human HER2 that showed different dynamics of HER2 *status*. MamBo89HER2^*stable*^ cell line displayed high and stable HER2 expression, which was maintained upon in vivo passages, whereas MamBo43HER2^*labile*^ cell line gave rise to HER2-negative tumors from which MamBo38HER2^*loss*^ cell line was derived. Both low-density seeding and in vitro trastuzumab treatment of MamBo43HER2^*labile*^ cells induced the loss of HER2 expression. MamBo38HER2^*loss*^ cells showed a spindle-like morphology, high stemness and acquired in vivo malignancy. A comprehensive molecular profile confirmed the loss of addiction to HER2 signaling and acquisition of an EMT signature, together with increased angiogenesis and migration ability. We identified PDGFR-B among the newly expressed determinants of MamBo38HER2^*loss*^ cell tumorigenic ability. Sunitinib inhibited MamBo38HER2^*loss*^ tumor growth in vivo and reduced stemness and IL6 production in vitro. In conclusion, HER2-positive mammary tumors can evolve into tumors that display distinctive traits of claudin-low tumors. Our dynamic model of HER2 *status* can lead to the identification of new druggable targets, such as PDGFR-B, in order to counteract the resistance to HER2-targeted therapy that is caused by HER2 loss.

## Introduction

Breast cancer is a major clinical problem and is the most frequent cause of death by tumor in women [1]. Breast cancer is not a single entity, but rather a multitude of diseases that include several subtypes with extremely different molecular, morphological and clinical characteristics [2]. In past decades, -omic techniques were used to define four intrinsic subtypes: luminal-A, luminal-B, HER2-enriched and basal-like. Nevertheless, a certain degree of molecular and clinical heterogeneity was found even within each intrinsic subtype [3].

HER2-enriched tumors account for 20–25% of breast cancers, and anti-HER2 targeted drugs significantly improved the survival rate of these patients [4]. However, HER2-positive breast cancer still presents a challenge for clinicians and researchers, mainly due to high rates of intrinsic and acquired resistance to HER2-targeted therapies, and brain metastasis [5].

HER2-expression heterogeneity can be identified at three different levels: (a) inter-tumor heterogeneity among tumors of different patients classified as HER2-positive; (b) inter-lesion heterogeneity between primary tumor and metastasis in the same patient; (c) intra-tumor heterogeneity within single lesions. Inter-tumor heterogeneity means that HER2-positive-classified tumors harbor a different number of HER2 copies in their DNA, or that they express the protein at different levels [6]. Second, a meta-analysis study has shown that the percentage of HER2 discordance between primary tumor and metastasis was 10.8%; HER2 changed twice as often from positive to negative (21.3%) than vice versa (9.5%) [7]. Lastly, sub-populations with different levels of HER2 expression can be identified as distinct clusters or interspersed cells in 1–40% of HER2-positive breast cancers [8].

At any level, HER2 heterogeneity is a clinical issue with a profound, but still-to-be-uncovered impact on prognosis. Several, but not all, studies have reported faster progression and a decrease in survival in patients whose metastases lose HER2 expression [9, 10]. Intra-tumor heterogeneity has been reported to have a detrimental effect on prognosis as well [8].

The success of HER2-targeted therapy is strictly related to the HER2 addiction of tumor cells [11]. Receptor conversion entails a risk of treatment failure in discordant metastasis [7]. Furthermore, the treatment of heterogeneous lesions with targeted drugs runs the risk of promoting the selection of target-negative sub-populations, and the consequent acquisition of resistance to targeted therapies [8, 12]. Anyway, the promotion of HER2 loss by trastuzumab neoadjuvant therapy is debated [10, 13].

In HER2-positive breast cancer, HER2 amplification is supposed to be the major driver of oncogenic transformation and tumor growth, but even HER2-negative elements can harbor alternative driver mutations. Epithelial-to-mesenchymal transition (EMT) seems to be involved in breast cancer progression and the ability of cells to switch between different phenotypes, known as epithelial-mesenchymal plasticity, is associated with resistance to targeted therapies [14].

Various strategies were employed to transiently inhibit HER2 expression in vitro and in vivo, and this unavoidably leads to the inhibition of cell and tumor growth [15]. Some studies reported the emergence of HER2-negative clones from HER2/neu-positive cell lines either under non-adherent growth conditions or upon continuous treatment with targeted drugs [16–18]. However, none of these works shed light on the underlying mechanisms.

This present work takes advantage of a preclinical model of in vivo HER2-expression loss and aims to identify alternative targets for the therapy of HER2-negative secondary lesions.

## Materials and methods

### Mice

FVBhuHER2-transgenic mice [19], obtained from Genentech Inc. (South San Francisco, CA, USA), were bred in our animal facility and genetically screened as previously reported [20]. BALB/c Rag2^-/-^;Il2Rg^-/-^ mice [21], were used as an immunodeficient model that lacked B, T, and NK immune components. Non-transgenic FVB female mice (FVB/NCrl) were purchased from Charles River Laboratories (Calco, Como, Italy). Blinding to assess the outcome of in vivo experiments was not done. For drug treatments, animals from each litter were allocated random to the different treatments. Five mice were enrolled in each test group in order to have an 80% chance of showing, with a 5% significance, a 65% of success in the experimental group.

All animal procedures were performed in accordance with European directive 2010/63/UE and Italian Law (DL26/2014); experimental protocols were reviewed and approved by the institutional animal care and use committee (“Comitato per il Benessere Animale”) of the University of Bologna and by the Italian Ministry of Health with letter 688/2015.

### Cell lines

MamBo cell lines were established from mammary tumors of FVBhuHER2 virgin female mice. Tumors were minced and set in culture with an appropriate medium as previously described [22]. Cell lines were stabilized and cultured in DMEM (Thermo Fisher Scientific, Monza, Italy) that was supplemented with 20% fetal bovine serum (FBS, Thermo Fisher Scientific), 30 µg/ml bovine pituitary extract (Corning Life Sciences, Glendale, AZ, USA) and 0.5% v/v MITO Serum Extender (Corning). Cell cultures were maintained at 37 °C in a humified 5% CO_2_ atmosphere.

### Tumorigenicity and metastatic ability of MamBo cell lines

To evaluate the tumorigenicity of the MamBo cell lines, FVBhuHER2 virgin female mice (12–20-week-old) received the injection of 10^6^ cells into the mammary fat pad (m.f.p.) (MamBo89HER2^*stable*^ cell line *n* = 8, MamBo43HER2^*labile*^ cell line *n* = 3, MamBo38HER2^*loss*^ cell line *n* = 5). Animals were inspected weekly to follow tumor development, which was measured with a caliper. Tumor volume was calculated as π/12*(√ab)^3^, where a = maximal tumor diameter and b=maximal tumor diameter perpendicular to a. Mice were sacrificed before tumors reached 2.5 cm^3^ or 10% of mouse weight. At necropsy, tumors were harvested and disaggregated with trypsin-0.05% EDTA (Thermo Fisher Scientific) for cytofluorimetric analysis.

Experimental metastatic potential was assessed in FVBhuHER2 virgin female mice (8–17-week-old) via the injection of 10^5^ cells into a caudal vein (*n* = 5). The general health status of the mice was checked weekly and the mice were either euthanized, as previously described, either at any sign of lung metastasis, or 18 weeks after cell injection. Lungs were stained with ink, fixed in Fekete’s solution and metastases were counted under a stereoscope.

To evaluate the dose-dependent tumor growth of MamBo43HER2^*labile*^ cells in immunocompetent mice, different doses of cells (10^6^, 10^7^ and 2 × 10^7^) were subcutaneously (s.c.) injected into FVBhuHER2 virgin female 11-week-old mice. MamBo43HER2^*labile*^ cells (10^5^ cells) were also s.c. injected into Rag2^-/-^;Il2Rg^-/-^ immunodeficient female 8-week-old mice (*n* = 3), compared to FVBhuHER2 virgin female 14-week-old mice (*n* = 3), to inspect the contribution of adaptive immunity to HER2 loss.

### Cytofluorimetric analysis

Harvested cells and tumor samples, which had previously been dissociated to yield single-cell suspensions, were analyzed by immunofluorescence and cytofluorimetric analysis as previously described [22]. The antibodies used for indirect immunofluorescence included: rat anti-mouse CD16-CD32 antibody Fc block (clone 2.4G2; diluted 1:100; BD Pharmingen, CA, USA); mouse anti-human HER2 (MGR2, diluted 1:100, Alexis Biochemical, Enzo Life Sciences, Lansen, Swisse) and also kindly provided by Dr. Elda Tagliabue (IRCCS, Istituto Nazionale dei Tumori, Milan, Italy); rat anti-mouse CD140b (PDGFR-B) (APB5, diluted 1:100; BioLegend, CA, USA). Anti-mouse IgGAF488 (diluted 1:100; Thermo Fisher Scientific) and anti-rat IgGFITC (diluted 1:40; KPL) were used as secondary antibodies. Direct immunofluorescence made use of: anti-human HER2PE (clone Neu 24.7, diluted 1:20, BD Pharmigen); anti-mouse CD24AF488 (clone M1/69; diluted 1:10; BioLegend); anti-mouse-CD44PE (clone IM7; diluted 1:10, BioLegend); anti-mouse Sca1PE (clone E13-161.7, 1:100 dilution; BioLegend); and anti-mouse CD29PE (clone HMβ1-1; diluted 1:10; BioLegend). Data were acquired using CyFlow Space, (Sysmex Partec, Germany) and analyzed using FCSExpress (De Novo Software, Glendale, CA, USA).

### Mammosphere formation assay

The ability of MamBo cell lines to form mammospheres in vitro was assessed using the MammoCult Human Medium Kit (STEMCELL Technologies, Vancouver, Canada), according to the manufacturer’s protocol (*n* = 4). Briefly, 4 × 10^4^ cells were seeded in 4 ml complete MammoCult medium without serum in 6 well UltraLow Adherence plates. Mammospheres that were bigger than 60 µm were counted on day 7. The same protocol was employed to evaluate the ability of MamBo38HER2^*loss*^ to form mammospheres in a medium that contained sunitinib 5 µM (vehicle and sunitinib *n* = 4; two tests with untreated cells were also run in parallel).

### Induction of HER2 loss in vitro and in vivo and trastuzumab treatment

MamBo43HER2^*labile*^ cells were kept in culture with trastuzumab (Herceptin, Roche) 120 h to evaluate the short-term growth inhibition and level of HER2 expression. A long-term culture, 2 months, was performed in the presence of 30 μg/ml trastuzumab. Cells were counted weekly and seeded at a concentration of either 1.6 × 10^5^ cells/cm^2^ (high density, also referred to as MamBo43HER2^*labile*^ TRT cells) or 4 × 10^4^ cells/cm^2^ (low density). In parallel, cells were harvested for molecular analysis, cytofluorimetric analysis of HER2 and stemness-marker expression. A third culture, without trastuzumab, was performed by seeding MamBo43HER2^*labile*^ cells at a lower density (10^4^ cells/cm^2^). Cells were split and harvested for cytofluorimetric analysis twice a week.

MamBo43HER2^*labile*^ cells (10^6^ cells) were s.c. injected into FVBhuHER2 virgin female mice (13–16-week-old) that were treated intraperitoneally (i.p.) with either saline (vehicle, *n* = 5) or trastuzumab 4 mg/kg twice a week starting from day 3 after cell injection (*n* = 5, a censored mouse at 2 weeks from cell injection was not included in tumor growth analysis).

### Sensitivity to demethylating agent

MamBo38HER2^*loss*^ cells were seeded at 8 × 10^4^ cells/cm^2^. After 24 h of seeding, cells were treated with vehicle (DMSO 0.02%), 5-aza-2′-deoxycytidine (Merck Life Science, Milano, Italy) 0.5 μM or 5 μM. Treatment was renewed after 72 h. Cells were harvested after 48, 72, and 144 h of treatment (from the first treatment) and counted. HER2 expression was determined by cytofluorimetric analysis.

### Sensitivity to sunitinib in vitro and in vivo

Sensitivity to sunitinib (sunitinib malate, LC Laboratories, MA, USA) was determined using the WST-1 reagent (Merck), according to the manufacturer’s instructions, on MamBo38HER2^*loss*^ cells, seeded at 3.125 × 10^3^ cells/cm^2^ in 96-well plates (Corning Life Sciences) and treated with sunitinib 0.1, 0.5, 1, 5, and 10 µM. Drug sensitivity was evaluated 72 h after treatment.

Sunitinib sensitivity in MamBo38HER2^*loss*^ cells (0.5 × 10^3^ cells/cm^2^) was also evaluated under 3D non-adherent conditions, as previously described [22]. Colonies were counted after 14 days.

The migratory ability of MamBo38HER2^*loss*^ cells in the presence of sunitinib was evaluated in a wound-healing test. Cells were seeded in 24-well plates in complete medium and allowed to sit until confluence. The cell monolayer was scratched with a pipette tip 200 μl, the medium was changed with or without sunitinib, 5 µM, and wound width was measured at times 0 and 24 h after scratching (untreated *n* = 8, vehicle *n* = 6, sunitinib 5 µM *n* = 8). Migratory ability was calculated as width (24 h)/width(t0)*100. FVBhuHER2 virgin female mice (8–16-week-old) that harbored tumors that were induced by the s.c. injection of MamBo43HER2^*labile*^ cells (10^6^ cells) were treated daily with sunitinib 60 mg/kg *per os* by gavage starting from 3 days after cell injection (*n* = 5, a censored mouse at 1 week from cell injection was not included in tumor growth analysis). Animals in the vehicle group received Methylcellulose 0.5%+Tween80 0.4% (all from Merck) (*n* = 5). FVB virgin female 8-week-old mice that harbored tumors that were induced by the s.c. injection of MamBo38HER2^*loss*^ cells (2.5 × 10^4^ cells) were treated daily with sunitinib 60 mg/kg *per os* by gavage starting from 1 day after cell injection (*n* = 5). Animals in the vehicle group received Methylcellulose 0.5%+Tween80 0.4% (*n* = 5).

### RNA-sequencing

Total RNA was extracted from cell pellets using Trizol Reagent (Thermo Fisher), according to the manufacturer’s instructions. RNA-seq libraries were generated using TruSeq RNA Sample Prep Kit v2 (Illumina), according to the manufacturer’s recommendations. High-throughput sequencing was carried out on a NextSeq 500 (Illumina) using 75 nucleotides, in single-end mode. Reads were analyzed on a SeqBox [23]. The generation of Demultiplexing (bcl2fastq Illumina tool version 2.17.1.14-2) counts using STAR (version 2.5) /RSEM (version 1.3.0), and differential gene expression analysis using DESeq2 (version 1.14.1, adjusted *P*-value < 0.1 and |log2 fold change | ≥1) were all performed within the SeqBox framework [23]. Two distinct analyses were performed on differentially expressed genes. Functional enrichment analysis was performed using the EnrichR web tool (https://maayanlab.cloud/Enrichr/) and protein-protein interactions were detected using the STRING database (www.string-db.org).

In the HER2-positive *vs* HER2-negative comparison, we also included samples from MamBo89HER2^*stable*^ cells and MamBo38HER2^*loss*^ cells that were treated long-term with trastuzumab for either 30 days (then w/o 30 days) or 60 days, both maintaining the initial shape, HER2 expression level and stemness profile of correspondent untreated cells. In detail, the HER2-positive samples included MamBo89HER2^*stable*^ parental cells, trastuzumab-treated MamBo89HER2^*stable*^ cells, a HER2-positive MamBo89HER2^*stable*^ clone and MamBo43HER2^*labile*^ cells. HER2-negative samples included MamBo38HER2^*loss*^ parental cells, trastuzumab-treated MamBo38HER2^*loss*^ cells and high density seeding MamBo43HER2^*labile*^ trastuzumab-treated (TRT) cells (30 days then w/o 30 days or 60 days) having lost HER2 expression. The comparison of HER2 stable *vs* HER2 labile cells only included MamBo89HER2^*stable*^ parental cell line and its HER2-positive clone, and MamBo43HER2^*labile*^ cells. For each sample were tested three replicates, at least (except for MamBo38HER2^*loss*^ trastuzumab-treated cells since the analysis included only two samples).

### IL6 production

Supernatants were collected from cells that were seeded 8 × 10^4^ cells/cm^2^ in medium that contained either sunitinib 5 µM (LC Laboratories, MA, USA) or DMSO 0.05% (here referred as vehicle) (Merck) or no drug. mIL6 production was analyzed using mouse IL-6 Quantikine ELISA Kit (R&D Systems, Minneapolis, MN, USA), according to the manufacturer’s protocol. The concentration of each sample was calculated by interpolating values on a standard curve. A stable IL6 producer mouse mammary cancer cell line (TS/A-IL6) was used as positive control of IL6 production [24].

### Real-Time PCR

RNA was extracted, quantified and reverse transcribed as previously reported [25]. cDNA was amplified using Sso Advanced SyBR Green Supermix (Bio-Rad Laboratories, CA, USA) reagents. Reactions were performed in a Thermal Cycler CFX96 (Bio-Rad Laboratories). Analyses were performed using Bio-Rad CFX Manager 3.1 Software, and relative quantification was calculated as ΔCt= Ct_gene_-Ct_housekeeping_. We used the following Bio-Rad assays: Cdh1 (qMmuCID0006332); Col3a1 (qMmuCID0006332); Col5a2 (qMmuCID0011413); Dsp (qMmuCID0019458); Fgfbp1 (qMmuCID0007813); Igfbp4 (qMmuCID0006155); Il1rn (qMmuCID0009153); Mmp2 (qMmuCID00021124); Ocln (qMmuCID0005446); Pdgfrb (qMmuCID0025167); Sparc (qMmuCID0023536); Vcan (qMmuCID0005235); Snai1 (qMmuCID0024342); Zeb1 (qMmuCID0009095); Zeb2 (qMmuCED0046769); Twist1 (qMmuCED0004065); and Ltbp1 (qMmuCED0045004). Custom HER2 primers were also used [26]. Mouse TBP ([27] or Bio-Rad assay qMmuCID0040542) was used as housekeeping gene.

DNA was extracted using a PureLink Genomic DNA Mini kit (Thermo Fisher Scientific), according to the manufacturer’s protocol. HER2 copy number was detected by Real-Time PCR using HER2 qHsaCEP0052301 assay (Bio-Rad Laboratories) and was normalized over human/mouse Ptger2 [28]. Amplification was performed using Sso Advanced Universal Probes Supermix (Bio-Rad Laboratories). The copy number of the human and murine cell lines was inferred by considering that MCF7 and MDA-MB-231 harbor 2 copies of HER2 in the genome. Human cell-line culture conditions have previously been reported [29].

### Western blot analysis

Protein extraction and Western blotting were performed as reported previously [20]. The following primary antibodies were used: anti-HER2 (clone 3B5, diluted 1:1000, Calbiochem, Merck), anti-pNeu (Tyr 1248) (diluted 1:1000, Santa Cruz Biotechnology, Santa Cruz, CA), anti-Stat3 (clone 124H6, diluted 1:1000, Cell Signaling, Danvers, MA), anti-pStat3 (clone D3A7, diluted 1:2000, Cell Signaling), anti-Actin (diluted 1:1000, Merck). Membranes were either incubated with polyclonal horse-radish-peroxidase conjugated anti-rabbit IgG antibody (diluted 1:3000, Bio-Rad Laboratories), or anti-mouse IgG antibody (diluted 1:1000, Santa Cruz Biotechnology). Protein presence was detected by chemiluminescent reaction (Bio-Rad Laboratories) before film exposure.

### Statistical analysis

Experimental in vitro conditions analyzed with statistical measures were repeated two times, at least. The number of samples was reported in the Materials and Methods and/or in the Figure Legend. Statistical analyses were performed through Prism 5 software (GraphPad software, La Jolla, CA, USA). The two-tailed unpaired Student’s *t*-test or t test with Welch’s correction were performed according to assumptions of the tests and the variance between the compared groups. The used test was reported in Figure Legend. All values are depicted as the mean and SEM.

## Results

### Loss of HER2 expression

**Fig. 1:**
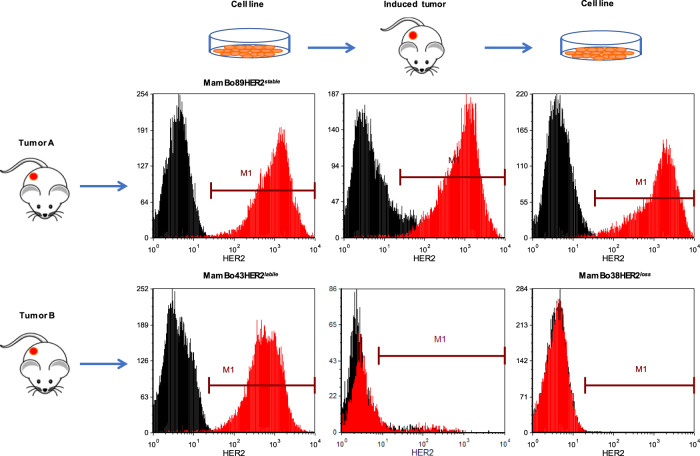
MamBo cell lines and dynamic HER2 expression. Panels show representative profiles of HER2 level as measured by cytofluorimetric analysis. Black profile, secondary antibody alone; red profile, anti-HER2 antibody.

FVB human HER2-transgenic (FVBhuHER2) mice develop spontaneous tumors with a heterogeneous expression of human HER2 [22]. Cell lines derived from tumors with a high HER2 level displayed different HER2-expression dynamics in vivo. We focused on two of these tumors to study cell behavior and involved mechanisms. Tumor A gave rise to the MamBo89HER2^*stable*^ cell line, which displayed high and stable HER2 expression that was maintained upon in vivo injection. Tumor B gave rise to the MamBo43HER2^*labile*^ cell line which, despite its comparably high expression of HER2, gave rise to HER2-negative tumors (Fig. 1 and Supplementary Fig. S1). The MamBo38HER2^*loss*^ cell line was derived from a tumor induced *via* the in vivo injection of the MamBo43HER2^*labile*^ cell line. MamBo38HER2^*loss*^ did not express HER2 on the cell surface (Fig. 1). No HER2-positive tumors were obtained from MamBo43HER2^*labile*^ cells that were injected in severely immunocompromised mice, lacking T, B and NK cells, proving that immune responses were not involved in HER2 loss (Supplementary Fig. S1).

### MamBo38HER2^loss^ cells show mesenchymal and stem-like properties

**Fig. 2:**
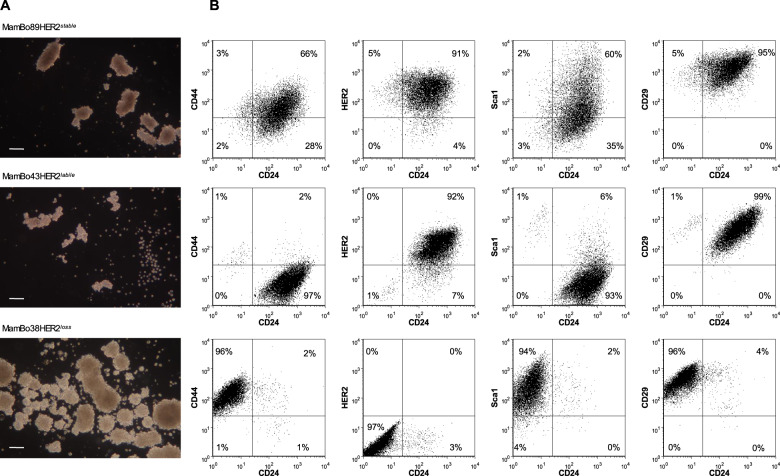
Stemness profile of MamBo cell lines. **A** Dark-field micrographs of mammosphere formation assay. White bar corresponds to 200 μm. Mean number of mammospheres ± SEM (*n* = 4): MamBo89HER2^*stable*^, 23 ± 1; MamBo43HER2^*labile*^, 17 ± 1; MamBo38HER2^*loss*^, 69 ± 9; MamBo38HER2^*loss*^ *vs* MamBo89HER2^*stable*^ and MamBo43HER2^*labile*^ cell lines, *p* < 0.01 by Student’s *t*-test. **B** Expression of HER2 and stemness markers CD24, CD44, Sca1 and CD29, in cells cultured under 2D-adherent conditions, measured by cytofluorimetric analysis.

The HER2-negative cell line, MamBo38HER2^*loss*^, displayed peculiarities compared to HER2-positive cell lines both in vitro and in vivo. MamBo89HER2^*stable*^ and MamBo43HER2^*labile*^ cell lines grew in vitro as monolayers of polygonal cells, whereas the MamBo38HER2^*loss*^ cell line formed a multilayer of spindle-like cells. The MamBo38HER2^*loss*^ cell line displayed a higher capacity to form mammospheres than MamBo89HER2^*stable*^ and MamBo43HER2^*labile*^ cell lines (Fig. 2A) together with highly stemness features: over 95% of cells were CD24^negative^/CD44^high^ and expressed high levels of Sca1 and CD29. The MamBo89HER2^*stable*^ cell line had a stemness profile with 3% of cells being CD24^low^/CD44^high^ and expressing other stemness markers at medium-high levels. On the contrary, MamBo43HER2^*labile*^ cells were mainly CD24^high^/CD44^negative^/Sca1^low^/CD29^high^, and hence not stemness, except for a tiny sub-population of cells (1%) that displayed stemness characteristics (Fig. 2B).

**Fig. 3:**
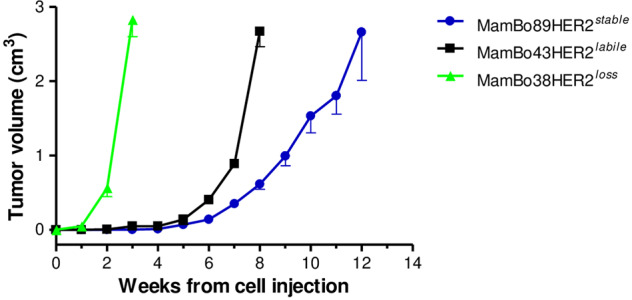
In vivo growth of MamBo cell lines (after injection of 10^6^ cells in m.f.p.) in FVBhuHER2 female mice. Cell lines: MamBo38HER2^*loss*^ (green triangle), MamBo43HER2^*labile*^ (black square) and MamBo89HER2^*stable*^ (blue circle). Mean and SEM from 3–8 mice per group is shown. MamBo38HER2^*loss*^ growth was significantly faster, from 1 week after cell injection onwards, than MamBo43HER2^*labile*^ and MamBo89HER2^*stable*^ cell lines, *p* < 0.01 by unpaired t test with Welch’s correction. From 6 weeks after cell injection, MamBo43HER2^*labile*^ cells also grew faster compared to the MamBo89HER2^*stable*^ cell line, *p* < 0.001 at 6-7 weeks and *p* = 0.068 at 8 weeks by unpaired t test with Welch’s correction.

Upon orthotopic in vivo injection, the MamBo38HER2^*loss*^ cell line displayed higher tumorigenicity than the HER2-positive cell lines (Fig. 3). Upon intravenous injection, the MamBo38HER2^*loss*^ cell line displayed the highest experimental metastatic ability and led to the complete substitution of lungs with metastatic nodules (>200) within 3 weeks. On the other hand, HER2-positive cell lines gave rise to few (MamBo89HER2^*stable*^ cells, median number of metastasis 2, range 0–4, and incidence 4/5 mice) or no (MamBo43HER2^*labile*^ cells) lung metastases 18 weeks after cell injection.

To sum up, the loss of HER2 expression in the MamBo38HER2^*loss*^ cell line was accompanied by spindle-like morphology, increased stemness and greater in vivo malignancy.

### Mechanisms of HER2 loss in vitro

Loss of HER2 expression in transgenic cell lines is not an unheard of evidence: we previously documented a similar phenomenon among cells obtained from transgenic mice harboring a rat HER2/neu gene [30]. The loss of HER2 expression in MamBo38HER2^*loss*^ cells could be the consequence of genomic events affecting the transgene. However, when we analyzed the HER2 copy number of MamBo38HER2^*loss*^ cells we found that it was similar to that of HER2-positive MamBo43HER2^*labile*^ cells (Supplementary Table S1), thus ruling out a total loss of transgene. We now plan to sequence the genome of this cells to determine whether the genes are intact.

The level of HER2 transcript was barely detectable, about 1000-times less in MamBo38HER2^*loss*^ cells as compared to MamBo89HER2^*stable*^ and MamBo43HER2^*labile*^ cells (Supplementary Fig. S2A).

Western blot analysis showed a complete lack of HER2 proteins within the cells (Supplementary Fig. S2B) thus excluding post-translational events.

To determine whether DNA methylation could be involved in the loss of HER2 expression we treated MamBo38HER2^*loss*^ cells with the DNA methil-transferase inhibitor 5-Aza-2’-deoxycytidine, however HER2 expression was not induced and the cells remained negative (Supplementary Fig. S3). We are currently studying whether other epigenetic modulators could be effective in unblocking HER2 expression in MamBo38HER2^*loss*^ cell line.

### Induction of HER2 loss in vitro

We investigated whether and how trastuzumab may affect HER2-expression loss in the MamBo43HER2^*labile*^ cell line.

Under adherent 2D-culture conditions, trastuzumab had little efficacy in inhibiting the growth of the MamBo43HER2^*labile*^ cell line (Supplementary Fig. S4A). Nevertheless, a cytofluorimetric analysis of trastuzumab-treated cells revealed an intriguing increase in the HER2-negative population (untreated 6% *versus* trastuzumab 16%) (Supplementary Fig. S4B).

**Fig. 4:**
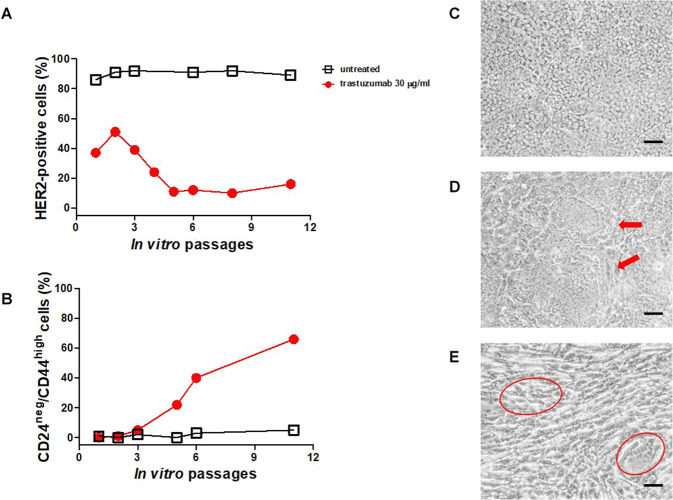
Phenotypic evaluation of MamBo43HER2^*labile*^ cells maintained in continuous culture with and without trastuzumab. **A**, **B** Cytofluorimetric analysis of cell culture at different time points in terms of number of in vitro passages. Black open square, untreated cells; red solid circle, cells cultured in presence of 30 µg/ml of trastuzumab. **A** Percentage of HER2-positive cells; **B** Percentage of cells with the staminal phenotype CD24^negative^/CD44^high^. **C**–**E** Micrographs of untreated cells after 4 in vitro passages **C** or trastuzumab-treated cells after 4 in vitro passages **D** and 8 in vitro passages **E**. Untreated cells are uniformly polygonal, whereas trastuzumab-treated cells show spindle-like area (indicated by the red arrows) after 4 in vitro passages. After 60 days of treatment (8 in vitro passages), the spindle-like population takes over and only a few islets of polygonal cells remain (indicated by the red circles). Black bar corresponds to 200 µm.

We therefore set up continuous in vitro culture with trastuzumab at 30 μg/ml for 2 months to verify whether prolonged exposure to trastuzumab can induce HER2-expression loss in the MamBo43HER2^*labile*^ cell line (Fig. 4). Untreated cells kept growing, over time and in vitro passages, as a monolayer of polygonal cells (Fig. 4C), whereas, in the presence of trastuzumab, an increasing subpopulation of spindle-like cells took over with only tiny islets of polygonal cells remaining interspersed within the multilayer after two months (Fig. 4E). The percentage of HER2-positive cells gradually diminished in trastuzumab-treated cells. However, a small percentage of HER2-positive cells (≤10%) was always detectable in the trastuzumab-treated population (Fig. 4A). In parallel with the reduction of the HER2-positive population, a sub-population with stemness characteristics (CD24^negative^/CD44^high^) was gradually expanded from an initial presence of less than 1% up to 65% after 2 months (Fig. 4B).

**Fig. 5:**
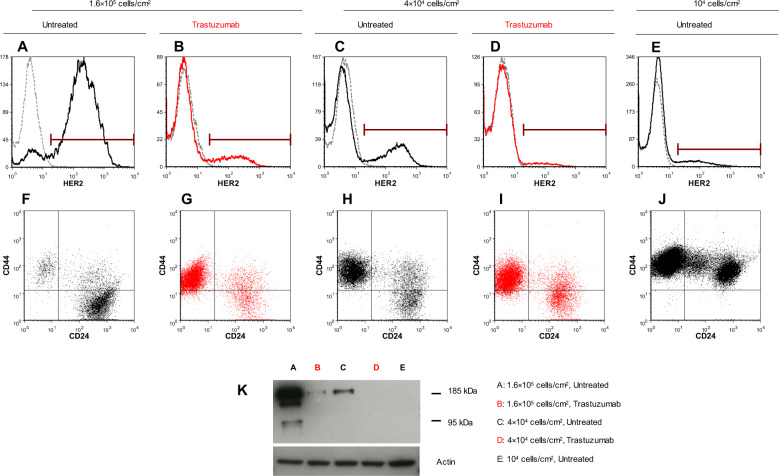
Effect of cell seeding and trastuzumab on phenotypic profile of MamBo43HER2^*labile*^ cells. Continuous cultures in control medium **A**, **C**, **E**, **F**, **H**, **J** or trastuzumab 30 µg/ml **B**, **D**, **G**, **I**. Level of HER2 **A**–**E** and stemness markers CD24 and CD44 **F**–**J** were measured by cytofluorimetric analysis. Cell seeding dose: 1.6 × 10^5^ cells/cm^2^, 60 days of culture **A**, **B**, **F**, **G**; 4 × 10^4^ cells/cm^2^, 60 days of culture **C**, **D**, **H**, **I**; 10^4^ cells/cm^2^, 30 days of culture **E**, **J**. **K** Western blotting analysis for HER2.

To understand whether the loss of HER2 expression was specifically related to trastuzumab treatment or whether it was an off-target consequence of any treatment that alters cell density, MamBo43HER2^*labile*^ cells were seeded at lower cell doses than in the previous experiment (4 × 10^4^ cells/cm^2^ *versus* 1.6 × 10^5^ cells/cm^2^) and again treated with trastuzumab at 30 µg/ml for two months (Fig. 5). Trastuzumab-treated cells acquired spindle-like morphology and the HER2-negative population took over (Fig. 5D). Moreover, 70% of the cells showed a stemness profile at the end of the long-term culture (Fig. 5I), as well as in high density cell seeding (Fig. 4 and Fig. 5B and G). Unexpectedly, even untreated cells spontaneously and gradually acquired spindle-like morphology at lower seeding density, and HER2 expression was detectable in less than 25% of cells after two months (Fig. 5C). At the same time, 70% of cells acquired a stemness phenotype (Fig. 5H). A further reduction in cell seeding dose (10^4^ cells/cm^2^) prompted the fast-track acquisition of the spindle-like morphology, the loss of HER2 expression and an enhancement in stemness within only one month, even in the absence of trastuzumab treatment (Fig. 5E and J). Cleaved forms of HER2 were not detected in cells with low/absent expression of full-length HER2 (Fig. 5K). Furthermore, we observed a significantly faster tumor growth of MamBo43HER2^*labile*^ cells in mice treated with trastuzumab than in untreated mice (Supplementary Fig. S4C). Overall trastuzumab appears to enhance the spontaneous trend towards the loss of HER2-expression in MamBo43HER2^*labile*^ cell line.

### Molecular portrait of HER2 dynamic expression

**Fig. 6:**
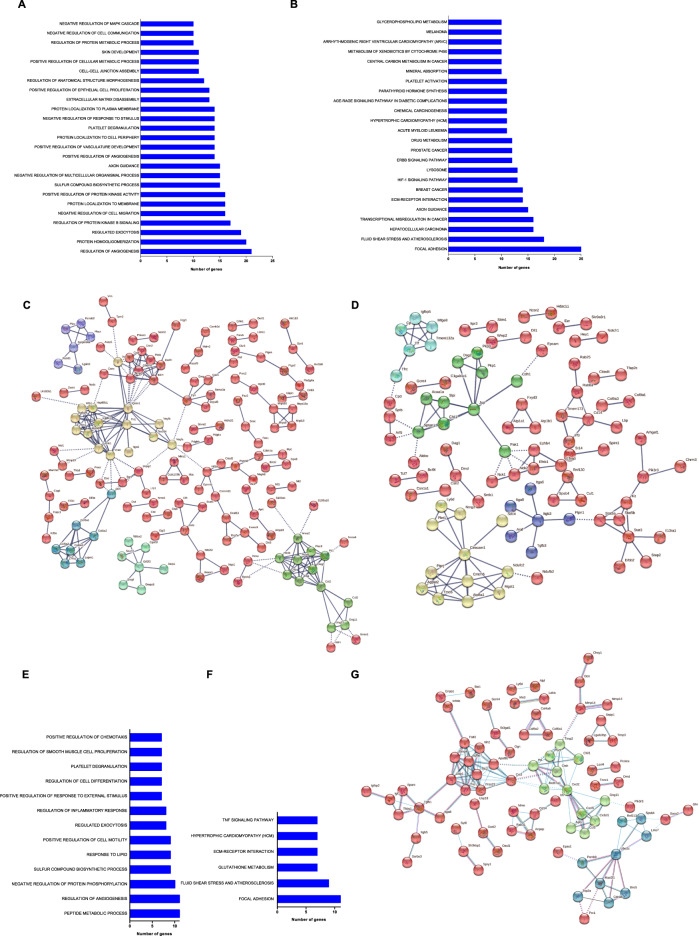
Differentially expressed genes in HER2-positive and HER2-negative cell lines **A**–**D** and in MamBo89HER2^*stable*^ versus MamBo43HER2^*labile*^ cells **E**–**G**. **A**, **E** Biological processes and **B**, **F** KEGG pathways with adjusted *p*-value<0.05, gathering genes >10 and excluding biological processes and pathways with a number of genes >200. **C**, **D** Representation of protein-protein interactions among HER2-negative up-regulated **C** and down-regulated **D** gene products (STRING, interaction confidence level 0.9, K-means cluster 5). **G** Representation of protein-protein interactions among MamBo89HER2^*stable*^ versus MamBo43HER2^*labile*^ differentially expressed genes *(*STRING, interaction confidence level 0.9, K-means cluster 3).

The transcriptomes of the cell lines that had lost HER2 expression, spontaneously in vivo or upon trastuzumab treatment in vitro, were compared to HER2-positive cell lines. Samples included in the comparison were listed in *Materials and Methods* section (subsection RNA-Sequencing). This analysis identified 751 differentially expressed genes: 402 were up-regulated in HER2-negative cell lines, whereas 349 were down-regulated. Differentially expressed genes were significantly associated into 25 different biological processes (Fig. 6A) and 24 KEGG pathways (Fig. 6B), which overall indicated a differential angiogenesis, migration ability, exocytosis, cell-cell adherence and communication and cellular differentiation. The up-regulated genes in HER2-negative cells are involved in extracellular matrix organization, the homo-oligomerization of proteins, angiogenesis and HIF-1 alpha signaling, exocytosis, the biosynthesis of compounds and cell signaling, and platelet activation. The down-regulated genes in HER2-negative cells are involved in protein localization to plasma membrane and cell periphery.

The main knots of interaction, analyzed by STRING, among genes products up-regulated in HER2-negative cells (Fig. 6C) are known to be down-regulated in HER2-positive breast cancer, or have, in turn, been associated with triple-negative breast cancers, such as Fn1 and Vim. Others, such as Vegf-a, Ptgs2, and Hif-α, were linked to the up-regulated pathway of angiogenesis. Furthermore, Dcn, Cav1, Cdkn1a, Myc, Qsox1, and Pdgfr-b are known for their role in promoting tumor-cell aggressiveness, EMT and sustaining the proliferation of mesenchymal cells. The EMT-associated profile of cells that lack HER2 expression was also confirmed by Real-Time PCR (Supplementary Fig. S5). Down-regulated genes in HER2-negative cell lines (Fig. 6D) are directly linked to HER2 overexpression and HER2-positive breast cancers (*e.g*., Stat, Ptpn1, Pak1, Efnb1). Other genes, such as Cecam1, Jup, Cdh1, Notch1, and Kit, are associated with polygonal shape and growth in a monolayer of closely interacting cells or luminal differentiation (Sptan1) of HER2-positive cell lines.

In order to identify the specific traits of tumor cells showing a tendency to lose HER2, we compared the molecular profiles of the MamBo89HER2^*stable*^ and MamBo43HER2^*labile*^ cell lines (Fig. 6E–G). The comparison highlighted 225 differentially expressed genes that are involved in metabolic processes, angiogenesis, biosynthetic processes, phosphorylation and cell motility. These genes clustered in pathways related to focal adhesion, glutathione metabolism, and TNF signaling pathway (Fig. 6E, F). STRING analysis evidenced networks of genes involved in cell plasticity (*e.g*. Tgfb1, Ltbp1, Bmp4, Dcn, and matrix metalloproteinases) (Fig. 6G).

### PDGFR-B as a target for HER2^loss^ cells

**Fig. 7:**
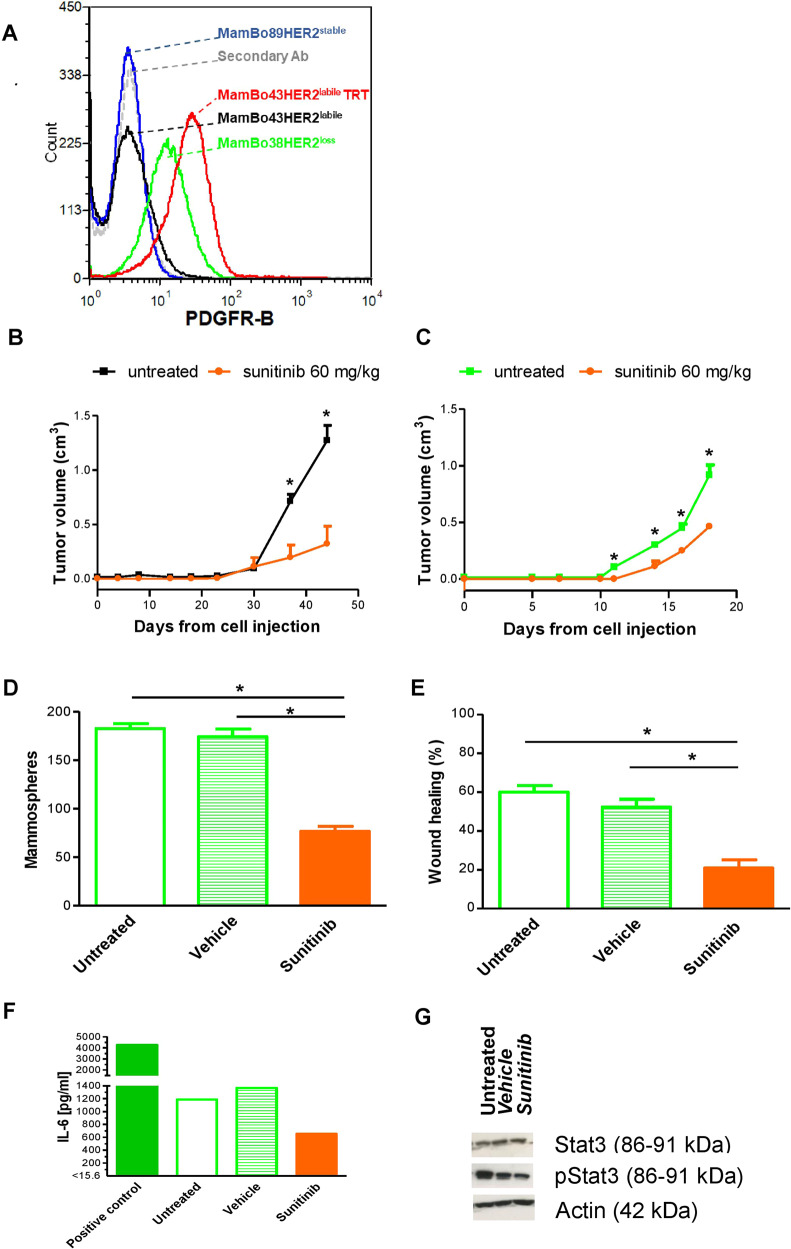
Targeting PDGFR-B in vivo (B-C) and in vitro (D-G). **A** Expression level of PDGFR-B by cytofluorimetric analysis. Profiles: gray, secondary antibody; blue, MamBo89HER2^*stable*^ cell line; black, MamBo43HER2^*labile*^ cell line; green, MamBo38HER2^*loss*^ cell line; red, trastuzumab-treated MamBo43HER2^*labile*^ cell line (MamBo43HER2^*labile*^ TRT). **B** Effect of sunitinib on MamBo43HER2^*labile*^ tumor growth. Sunitinib significantly reduced tumor growth from the 37^th^ day after cell injection onward, *p* < 0.05, at least, by unpaired t test with Welch’s correction. **C** Effect of sunitinib on MamBo38HER2^*loss*^ tumor growth. Sunitinib significantly reduced tumor growth from the 11^th^ day after cell injection onward, *p* < 0.05, at least, by unpaired t test with Welch’s correction. In **B**, **C** untreated mice (black square, MamBo43HER2^*labile*^ or green square, MamBo38HER2^*loss*^) or 60 mg/Kg sunitinib-treated mice (orange circle). Data shown are the mean and SEM from 4–5 mice per group. **D**–**G** Effect of sunitinib (5 μM) on MamBo38HER2^*loss*^. **D** Mammosphere formation assay. Data shown are the mean and SEM, *n* = 2–4 for each group; *p* < 0.001, sunitinib vs untreated or vehicle by Student’s t-test. **E** Wound-healing assay. Data shown are the mean and SEM, *n* = 6–8 for each sunitinib-treated group. *p* < 0.001, vs untreated or vehicle by Student’s t-test. **F** IL6 production detected by ELISA. **G** Western blotting analysis for STAT3 and pSTAT3 on cells treated with sunitinib.

Molecular analysis (Figs. 6C, 7A and Supplementary Fig. S5) indicated that PDGFR-B may be a molecule that sustains the growth of HER2-negative cell lines. This hypothesis grew stronger upon analyzing the list of genes that are up-regulated in HER2-negative cell lines using the ArchS4Kinase database; at least 50 genes were found to be significantly associated with the kinase activation of PDGFR-B (Supplementary Table S2).

Under 3D non-adherent conditions, sunitinib 1 µM dramatically reduced the size of the colonies, but colony count was reduced by only 20%. Sunitinib at 5 µM completely impeded the growth of any colonies (Supplementary Fig. S6B). Sunitinib efficacy in reducing the aggressiveness of HER2-negative cells was confirmed in vivo, where it significantly slowed down tumor growth induced by cell injection of MamBo43HER2^labile^ (Fig. 7B) and MamBo38HER2^loss^ cells (Fig. 7C).

Sunitinib modified two distinct traits of MamBo38HER2^loss^ cells in vitro. First, treatment reduced mammosphere formation by 50%, as well as migration ability (Fig. 7D, E). Furthermore, 2D-treated cells mostly acquired a more polygonal shape, and the cells appeared to grow in a monolayer with fewer spindle-like cells on a different focus (Supplementary Fig. S6C). This morphological change was associated with a slight modulation of E-cadherin expression (Supplementary Fig. S6D). It is worth noting that sunitinib reduced IL-6 production (Fig. 7F) and inhibited its downstream pathway through down-modulation of pStat3 (Fig. 7G).

## Discussion

Heterogeneity is a major clinical challenge in cancer. Over the last twenty years, a great deal of effort has gone into distinguishing distinct breast cancer subtypes using specific molecular signatures [31–35]. Nevertheless, intrinsic subtypes conceal further heterogeneity [36]. Breast cancers that are classified as HER2-positive include tumors with very different levels of HER2 amplification and overexpression [37, 38], and some HER2-overexpressing tumors may fall into other intrinsic subtypes when molecular signatures are considered [39]. Other than HER2 itself, its isoforms, *i.e*. Delta16 and p95HER2, may be present at different levels and ratios, further complicating the scenario [40, 41]. The tumors developed in FVBhuHER2 mice recapitulated HER2 inter-tumor heterogeneity, as found in clinic and other preclinical models, such as HER2-positive breast cancer cell lines of human origin [42, 43] and breast cancer PDX collections [44, 45].

Inter-lesion heterogeneity in a single patient is also a clinical issue. A pooled meta-analysis has reported a 10.8% HER2 discordance between primary tumor and metastases, and it was observed that HER2 changed twice as often from positive to negative (21.3%) than vice versa (9.5%) [7, 46, 47]. The effect of receptor conversion on prognosis is controversial. BRITS and DESTINY clinical trials did not show that receptor conversion had a significant effect on patient survival [46], whereas other studies have reported a decreased time to progression and survival in patients that lose HER2 expression in metastasis [9, 10, 13, 16].

We have reported a spontaneous model of HER2 loss in which the loss of HER2 expression is associated with a spindle-like morphology, increased IL-6 production, a gene expression profile that is reminiscent of EMT, increased stemness features and malignancy in vivo. Cell density in in vitro cultures influenced the loss of HER2 expression as previously seen in cell lines that were derived from mammary tumors from rat activated HER2/neu (NeuT) transgenic mice, where density considerably influenced the expression of neu and/or EMT traits [48]. It is reasonable to think that any anti-tumor treatment diminishes cell density in tumor mass and could lead to the emergence of HER2-negative populations in HER2-positive lesions.

The main issue for the success of HER2-targeted therapies is to ensure that most tumor cells are indeed addicted to HER2 expression for the maintenance of the malignant phenotype [11]. In the case of intra-tumor heterogeneity, with HER2-positive and HER2-negative sub-clones, it is likely that targeted therapies will clear off HER2-positive cells, but not eradicate the tumor, which will relapse as HER2-negative. The literature contains contrasting data on the effect of neoadjuvant and adjuvant therapy with trastuzumab on promoting the loss of HER2 expression in metastasis [16, 49]. Moreover, some papers have reported that prolonged anti-HER2 treatment promotes the emergence of HER2-negative, spindle-like, highly aggressive and stem cells from HER2/neu positive cell lines both in vitro and in vivo [16–18, 50]. In our model, the addition of trastuzumab treatment to low-density MamBo43HER2^*labile*^ in vitro, or upon its in vivo injection, appears to accelerate the spontaneous loss of HER2 expression and the emergence of highly stemness and aggressive populations.

The mechanisms that underlie receptor conversion are mainly unknown due to the limited amount of information that is available on the molecular profiles of these patients and the paucity of preclinical models that can reproduce this condition. The inactivation of HER2 has been invariably associated with either tumor regression or tumorigenicity loss [15–18, 30, 51]. The loss of tumorigenicity upon HER2 loss or experimental inhibition is in line with the theory of “oncogene addiction” [52]. By contrast, our preclinical model of spontaneous and trastuzumab-induced HER2 loss is associated with increased stemness and a greater aggressiveness in vivo. Thus, it is also conceivable that, in our model, further oncogenic transformations occurred that permitted HER2 addiction to be overcome and the malignant HER2-negative phenotype to be sustained.

The molecular profile of the HER2-negative cells in our model mirrored that of breast cancers that lack HER2 expression [53–56]. Gene expression also reflected the functional differences between HER2-negative and HER2-positive cell lines. The EMT and stemness profile of HER2-negative cells resemble the peculiar traits of claudin-low tumors, which are defined as tumors with low expression of cell-cell adhesion genes, high expression of EMT genes and stem-cell-like/less differentiated gene expression patterns [39]. Fougner and colleagues [57], have recently redefined claudin-lowness as a condition that is present in various intrinsic subtypes, rather than in a distinct subtype. This observation led us to hypothesize that HER2-positive primary lesions can progress through acquisition of claudin-lowness.

In the search for possible pathways that sustain the malignant phenotype of HER2-negative cells that have lost their addiction to HER2, we identified PDGFR-B. PDGFR-B is known to sustain cancer progression by promoting EMT and stemness phenotype [58, 59]. This molecule is also a pericyte marker and its expression on tumor cells with a mesenchymal phenotype suggested these cells have a role in angiogenesis as pericyte-like cells [60]. PDGFR-B is a druggable target by sunitinib. This multi-targeted molecule offers the possibility of inhibiting VEGFR, which was also identified as being up-regulated in HER2-negative cells in our analysis. Sunitinib was effective in halting the growth of MamBo38HER2^*loss*^ cells and the emergence of HER2-negative tumors from MamBo43HER2^*labile*^ cells. Moreover, we observed a transient loss of mesenchymal features in favor of epithelial traits at both the morphological and genetic levels, in sunitinib-treated cells, and this may reflect the existence of an epithelial/mesenchymal intermediate state [61]. In our model, IL6 production was detected in MamBo38HER2^*loss*^, and treatment with sunitinib lowered its production. IL6 up-regulation has been reported in a model of long-term-trastuzumab-treated BT474/PTEN^-/-^, which became spindle-like upon this treatment; IL6 appeared to trigger an inflammatory loop, which led to the acquisition of a stemness, basal-like phenotype and resistance to trastuzumab [62]. The efficacy of sunitinib as an anti-EMT target therapy has been proven in claudin-low human breast cancer cell lines [63], and this drug may be able to take advantage of IL6 inhibition.

Taken together, these data indicate the putative efficacy of the therapeutic targeting of PDGFR-B by sunitinib in HER2-negative cells. Nevertheless, treatment with sunitinib did not eradicate HER2-negative tumors, thus indicating that the PDGFR-B signaling pathway clearly sustains the growth of HER2-negative cells but is likely not the only driver of their malignancy. Hence, it might be interesting to investigate other targets, such as MYC, in HER2-negative cells.

HER2-positive tumors can show heterogeneous HER2 expression and heterogeneous behavior, whatever due to clonal selection or EMT, for this reason additional driver mutations should be investigated and taken into account for targeted therapies able to hamper tumor progression.

## Conclusions

We have proposed a dynamic model of HER2-positive mammary tumors that tends to spontaneously lose HER2 expression and progress towards a more aggressive phenotype via the acquisition of EMT and stemness properties, which are distinctive traits of claudin-low tumors.

Spontaneous loss of HER2 expression was affected by cell density and accelerated by treatment with trastuzumab. How cellular density manages to influence HER2 expression, *e.g*. via soluble factor concentration or molecules involved in cellular junction, will require further investigation.

Finally, our dynamic model of HER2 *status* is a worthwhile mean for the detection of druggable targets that may counteract resistance to HER2-targeted therapy due to HER2 loss. We have identified PDGFR-B as a possible target and proved the ability of sunitinib in delaying growth of tumors that evolved from HER2-positive to HER2-negative *status*. These results pave the way for the use of sunitinib in the treatment of patients with HER2 receptor conversion.

## Supplementary information

Supplementary materials

## Data Availability

RNA-Sequencing data can be found in Gene Expression Omnibus, GEO accession number: “GSE181468”.
