# Supplementary material for: Evolution of HER2-positive mammary carcinoma: HER2 loss reveals claudin-low traits in cancer progression
Source: Oncogenesis. 2021 Nov 13;10(11):77. doi: 10.1038/s41389-021-00360-9 (PMC8590694; doi:10.1038/s41389-021-00360-9)
Supplement: Supplementary file 1 — Supplementary materials [file 41389_2021_360_MOESM1_ESM.pdf]

Evolution of HER2-positive mammary carcinoma: HER2 loss reveals claudin-low traits in cancer progression

Veronica Giusti[1, #], Francesca Ruzzi[2, #], Lorena Landuzzi[1, #], Marianna L. Ianzano[2], Roberta Laranga[2], Elena Nironi[2], Laura Scalambra[2], Giordano Nicoletti[1], Carla De Giovanni[2], Martina Olivero[3, 4], Maddalena Arigoni[4], Raffaele Calogero[4], Patrizia Nanni[2, *], Arianna Palladini[2,5 $] and Pier-Luigi Lollini[2, $, *]

Supplementary Table S1. Number of huHER2 gene copies quantified by Real-Time PCR. $\Delta Ct = Ct_{huHER2} - Ct_{hu/mPTGER2}$. Expression level of $2^{-(\Delta ct\ MDA\text{-}MB\text{-}231 + \Delta ct\ MCF7)/2}$ was associated to two HER2 copies (n=2, at least). TS/A is a murine mammary cancer cell line.

Cell line	ΔCt	huHER2 copy number
Mambo89HER2*stable*	-7.27	51
Mambo43HER2*labile*	-5.83	19
Mambo38HER2*loss*	-5.79	18
TS/A	7.26	0
Non-transgenic normal tissue	4.25	0
HER2-transgenic normal tissue	-5.96	26
HER2-transgenic mammary tumor	-6.60	32
HCC1954	-9.34	215
BT474	-7.53	61
SKBr3	-5.25	13
MDA-MB-453	-4.14	6

Supplementary Table S2. List of kinases with significantly co-expressed genes among those up-regulated in HER2-negative cells as analysed by ArchS4 Kinase.

Term	Overlap	Adjusted P-value
DDR2	51	8.76E-30
PDGFRB	50	4.94E-29
PDGFRA	48	3.88E-27
ACVR1	47	2.42E-26
MYLK	47	2.42E-26
RPS6KA2	46	2.04E-25
NEK7	40	1.03E-19
LATS2	38	5.90E-18
MERTK	33	9.25E-14
SRC	33	9.25E-14
GRK5	30	1.89E-11
NEK6	30	1.89E-11
ABL2	29	1.01E-10
IRAK2	28	4.90E-10
CAMK1	28	4.90E-10
MAP3K6	27	2.46E-09
DYRK3	26	1.19E-08
PTK7	24	2.22E-07
CSF1R	25	5.52E-08
SGK1	24	2.22E-07
TGFBR2	24	2.22E-07
ACVRL1	23	8.39E-07
FGFR1	23	8.39E-07
TRIO	23	8.39E-07
ABL1	23	8.39E-07
ALPK2	22	3.21E-06
ILK	22	3.21E-06
LIMK1	22	3.21E-06
TEK	21	1.22E-05
SIK2	21	1.22E-05
DAPK1	20	4.57E-05
CDK15	19	1.49E-04
DAPK3	19	1.49E-04
MET	19	1.49E-04
PKDCC	19	1.49E-04
AXL	19	4.64E-04
RIPK1	18	4.64E-04
MAPK13	18	4.64E-04
EGFR	18	4.64E-04
MAP3K8	18	1.38E-03
PLK3	17	1.38E-03
RYK	17	1.38E-03
CDK4	17	1.38E-03
TIE1	17	3.74E-03
IKBKE	16	3.74E-03

Term	Overlap	Adjusted P-value
PIM3	16	3.74E-03
CDC42BPB	16	3.74E-03
EPHA2	16	3.74E-03
MAP2K3	16	3.74E-03
RIPK2	16	1.02E-02
MAP4K4	15	1.02E-02
PLK2	15	1.02E-02
RNASEL	15	2.63E-02
VRK2	14	2.64E-02
ROR2	14	2.62E-02

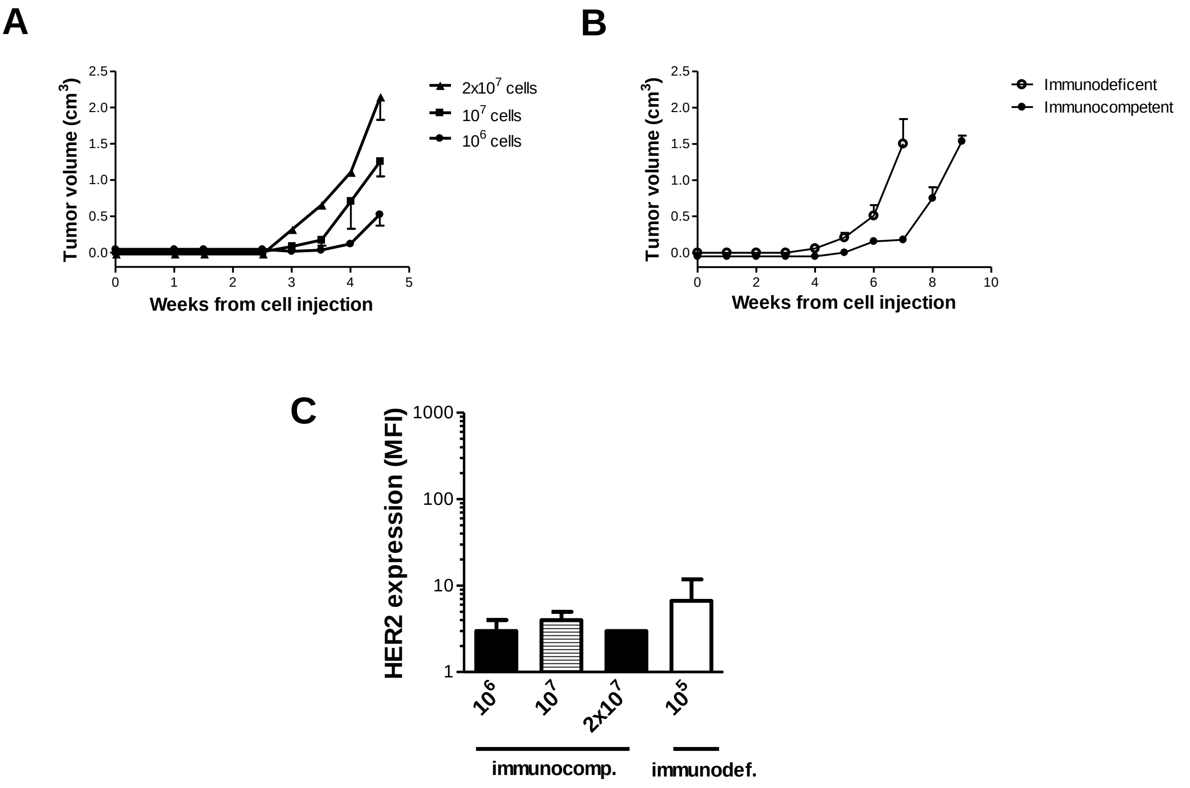

Supplementary Fig. S1. Tumor growth of s.c. injected MamBo43HER2^{labile} cells in different *in vivo* models. (A) MamBo43HER2^{labile} cells were s.c. injected into FVBhuHER2 mice at different doses: 10^6 (circle), 10^7 (square), $2x10^7$ (triangle). (B) MamBo43HER2^{labile} cells were s.c. injected (10^5 cells) into immuno-deficient mice (open circle) or FVBhuHER2 immunocompetent mice (solid circle). Data showed mean and SEM of 2-3 mice. (C) HER2 expression in tumors growing in immunocompetent mice as in picture (A) and in immunodeficient mice as in picture (B) detected by cytofluorometric analysis. Each bar represents the mean and SEM of 2-3 tumors.

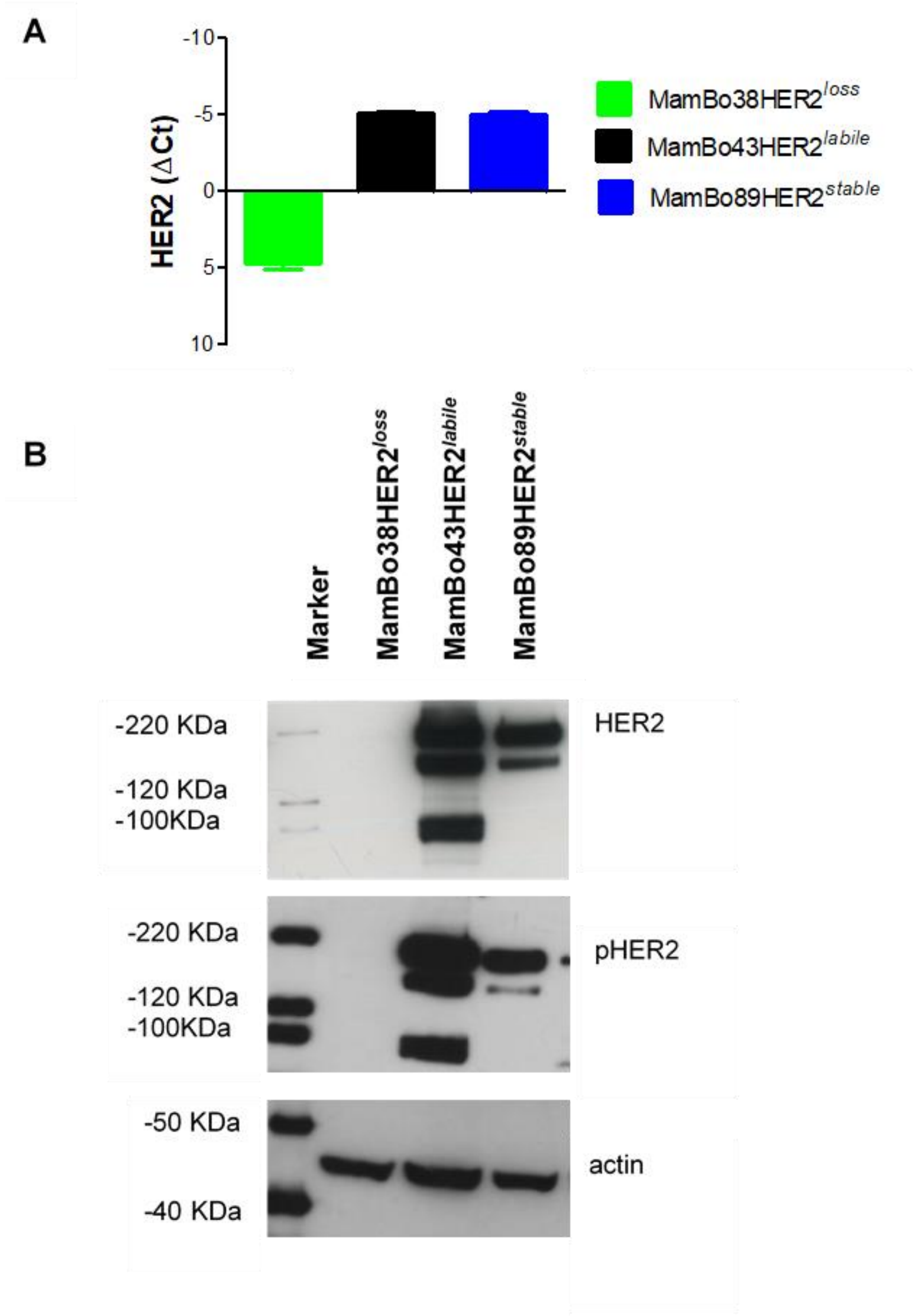

Supplementary Fig. S2. HER2 expression by MamBo cell lines. (A) Level of HER2 transcript by Real-Time PCR; MamBo89HER2^{stable} and MamBo43HER2^{labile}, n=2; MamBo38HER2^{loss}, n=4. Each bar shows mean and SEM. $\Delta Ct = Ct_{huHER2} - Ct_{mTBP}$ (B) Levels of HER2 protein and of its phosphorylated isoform pHER2 detected by Western blot.

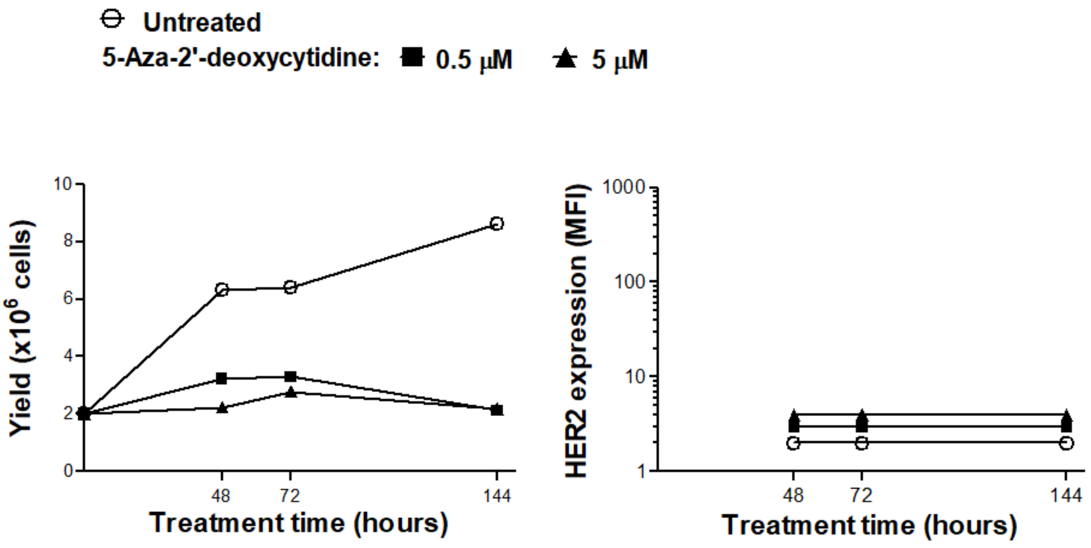

Supplementary Fig. S3. Effect of demethylation on MamBo38HER2*loss* cell line. Cells were seeded at $8x10^4$ cells/cm^2 and treated with 5-aza-2′-deoxycytidine 0.5 μM (square) or 5 μM (triangle) or medium only (circle). (A) Cell growth. (B) HER2 expression evaluated by flow-cytometry and reported as fluorescence intensity (MFI).

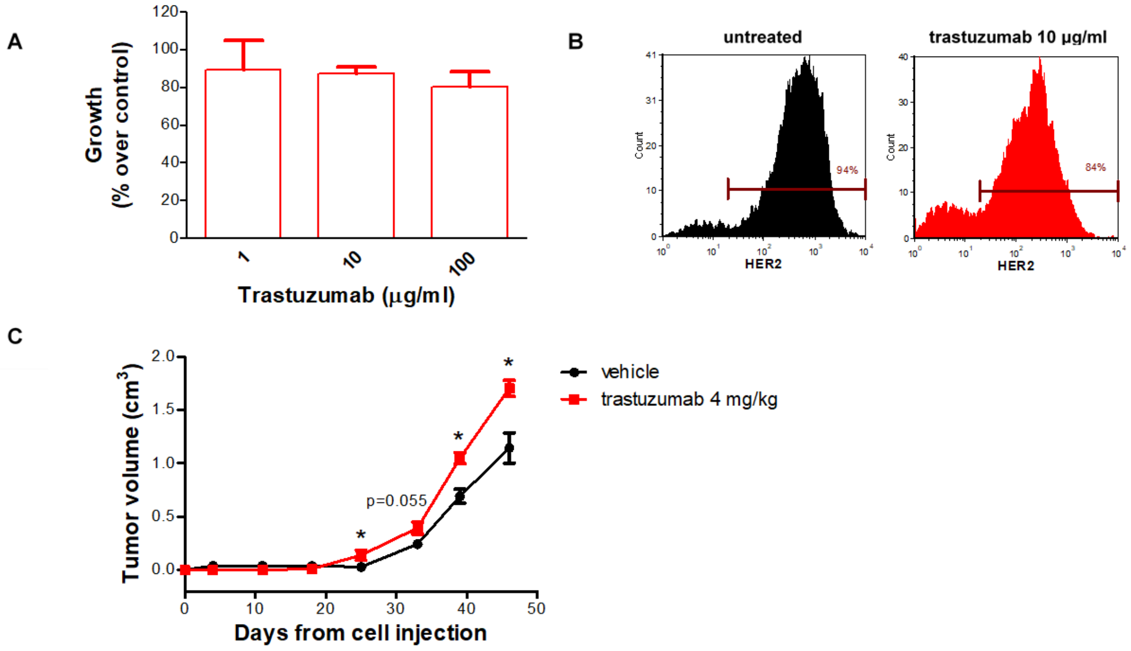

Supplementary Fig. S4. Trastuzumab treatment in MamBo43HER2^{labile} cells *in vitro* and *in vivo*. (A) Sensitivity on 2D culture after 120h of treatment. Each bar showed the mean and SEM; n=4. (B) HER2 expression of untreated (black profile) and trastuzumab-treated (10 µg/ml) (red profile) cells measured by cytofluorometric analysis. (C) Trastuzumab effect *in vivo* (4 mg/Kg) on the growth of tumors induced by subcutaneous injection of MamBo43HER2^{labile} cells. Vehicle, black circles; trastuzumab 4 mg/Kg, red squares; n=4-5 mice. *p<0.05 *vs* vehicle, by Student's *t*-test.

.

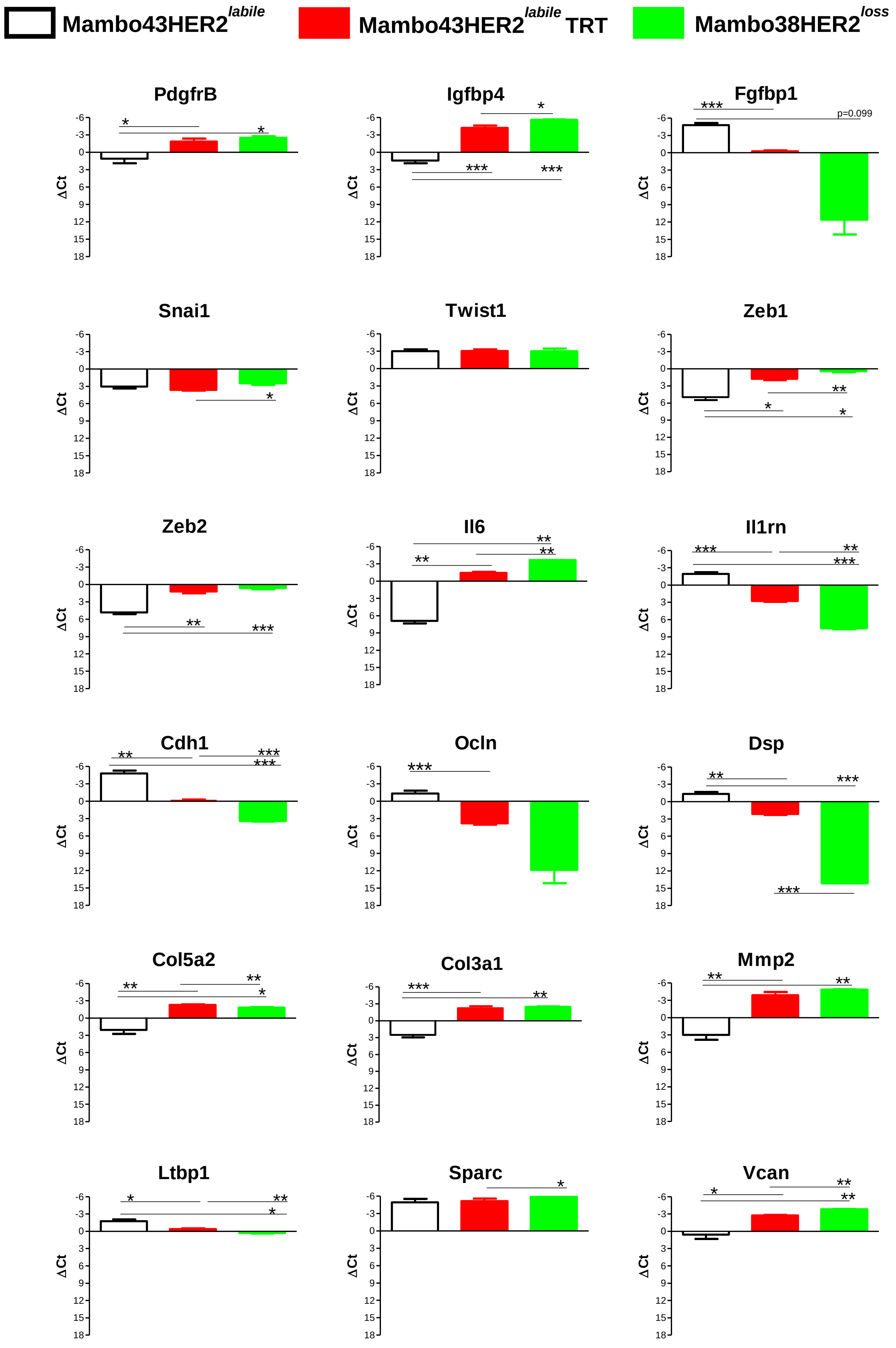

Supplementary Fig. S5. EMT profile by Real-Time PCR of MamBo43HER2*labile*, trastuzumab-treated MamBo43HER2*labile* (MamBo43HER2*labile* TRT) and MamBo38HER2*loss* cell lines respectively n=3, n=4 and n=2, at least. $\Delta Ct = Ct_{huHER2} - Ct_{mTBP}$. Each bar shows mean and SEM. *p<0.05; **p<0.01; ***p<0.001, by unpaired t test with Welch's correction.

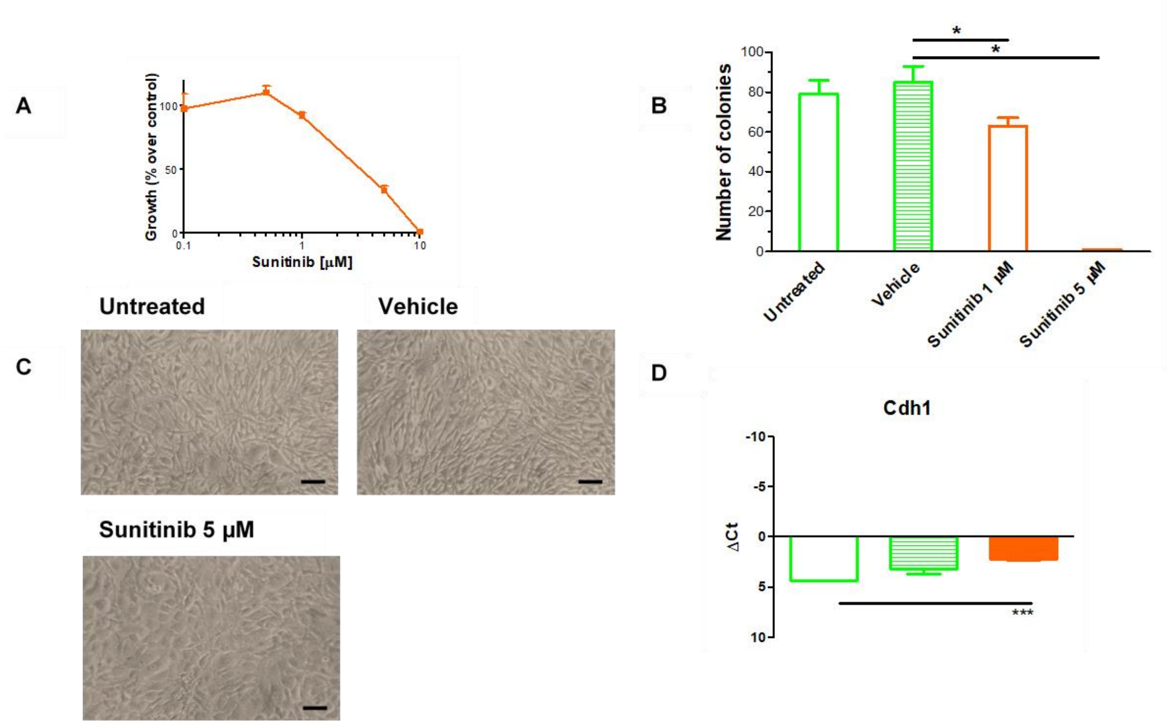

Supplementary Fig. S6. Sunitinib activity *in vitro* on MamBo38HER2^{loss} cells. (A) Growth under 2D-adherent conditions. n=3. (B) Growth under 3D non-adherent conditions of sunitinib-treated cells (1 and 5 μM). * p<0.05 versus vehicle. Respectively, by Student's *t*-test. n=4 (C) Sunitinib (5 μM) modified morphology of MamBo38HER2^{loss} cells. Bar is equivalent to 200 μm. (D) E-cadherin expression by Real-Time PCR of untreated (green), vehicle (green with rows) and sunitinib-treated (5 μM) cells (solid orange). Data reported mean and SEM. $\Delta Ct = Ct_{huHER2} - Ct_{mTBP}$. n=2. *p<0.05 by Student's *t*-test.
